# An Examination of the Generalizability of Motor Costs

**DOI:** 10.1371/journal.pone.0053759

**Published:** 2013-01-14

**Authors:** Max Berniker, Megan K. O’Brien, Konrad P. Kording, Alaa A. Ahmed

**Affiliations:** 1 Department of Physical Medicine and Rehabilitation, Northwestern University and Rehabilitation Institute of Chicago, Chicago, Illinois, United States of America; 2 Department of Mechanical Engineering, University of Colorado Boulder, Boulder, Colorado, United States of America; 3 Department of Integrative Physiology, University of Colorado Boulder, Boulder, Colorado, United States of America; The University of Western Ontario, Canada

## Abstract

Most approaches to understanding human motor control assume that people maximize their rewards while minimizing their motor efforts. This tradeoff between potential rewards and a sense of effort is quantified with a cost function. While the rewards can change across tasks, our sense of effort is assumed to remain constant and characterize how the nervous system organizes motor control. As such, when a proposed cost function compares well with data it is argued to be the underlying cause of a motor behavior, and not simply a fit to the data. Implicit in this proposition is the assumption that this cost function can then predict new motor behaviors. Here we examined this idea and asked whether an inferred cost function in one setting could explain subject’s behavior in settings that differed dynamically but had identical rewards. We found that the pattern of behavior observed across settings was similar to our predictions of optimal behavior. However, we could not conclude that this behavior was consistent with a conserved sense of effort. These results suggest that the standard forms for quantifying cost may not be sufficient to accurately examine whether or not human motor behavior abides by optimality principles.

## Introduction

Many, if not most, approaches to modeling human motor behavior are based on an assumed tradeoff between the potential rewards and efforts of a behavior. For example, moving can result in potential rewards, such as obtaining food, avoiding a hazard, or any number of other gains. However, to move we must exert our muscles. This requires effort, which may be judged by calories consumed, muscle activity, force generated, fatigue, or any number of similar expenses. While the rewards for any motor behavior can change from task to task, the effort associated with a specific motor behavior should remain constant. That is, grasping a bowl of cherries and grasping a bowl of pits constitute different rewards, but the effort of moving your arm should not depend on what you have seized.

Optimal control is a prominent framework for modeling human motor behavior[1]–[3]. Within this framework, the tradeoff between effort and reward is quantified by a candidate cost function. Behaviors that optimize this cost function can be computed using sophisticated mathematical techniques. These optimal behaviors are then compared with actual human behavior. Using this approach, a wide range of behavioral patterns can be explained by proposing subjects minimize relatively simple cost functions [4]–[21]. The ultimate value of using this optimal control framework, however, is that identifying a cost function used by the nervous system will illuminate how motor behaviors in general are organized.

Motor behaviors may be categorized by the properties of their dynamics (linear or nonlinear) and their degree of uncertainty (deterministic or stochastic). While many studies have investigated if motor behaviors optimize various senses of effort in each of these settings, they have been confined to a specific setting; e.g. linear stochastic or nonlinear deterministic settings. Despite the assumption that candidate costs should generalize, little is known about the ability of a proposed sense of effort to make predictions that generalize across these settings. Yet, if the nervous system minimizes a consistent sense of effort, then we should be able to predict motor behaviors in any setting if the rewards are known. If this sense of effort does not generalize, then it is not clear whether these costs are the causes of motor behavior, or simply descriptive of them.

To examine how well this sense of effort generalizes, we designed a motor task where the settings changed, but the reward remained the same. Accordingly, a subject’s inferred cost function was implicitly a fit to his or her sense of effort, relative to the task’s reward structure. If a subject’s sense of effort remained constant, then their overall cost function would generalize across the different settings. Hence, by inferring this cost function in one setting, we should be able to predict subject behavior in a different setting if this sense of effort generalized. Using this logic, our experimental task was designed such that the behaviors in each setting would differ from each other in relatively small but distinct ways. This helped ensure the fit cost functions would still be valid if subjects used a conserved sense of effort, yet differ enough to allow for non-trivial predictions.

Across the great majority of optimal control studies, a cost function is proposed in terms of quadratic penalties on state and/or task variables. For instance, the squared value of jerk [22], squared torque rate [6], or the squared error from a target [23] are common proposals. In part this is done for mathematical convenience, as analytical solutions exist for this class of problems. A quadratic cost is also equivalent to the Taylor expansion of a higher-order nonlinear cost for small deviations in state around a nominal optimal solution [24], [25]. Additionally, a quadratic penalty was shown to closely approximate the actual measured cost function on movement error [26]. As such, the choice of quadratic cost functions to characterize motor effort is often reasonable for a wide range of models and motor behaviors, and one we made here.

We had subjects perform a motor task with invariant rewards in 3 different settings: linear, nonlinear, and nonlinear stochastic. In all settings the goal was the same: to steer a virtual mass towards a target. In the linear setting, subjects guided the mass by isometrically pushing or pulling a handle. In the nonlinear setting, the subjects had to guide the mass towards the target in the presence of a virtual cliff. If the mass fell below the cliff, a virtual gravity force would pull it downwards requiring the subjects to produce large forces to get the mass back on target. In the nonlinear stochastic setting, there was a virtual cliff and random noise perturbing the mass’s trajectory. We found that the optimization of a quadratic cost inferred from the linear setting could predict the qualitative trends of subject behavior in the other two settings. However, the results precluded us from concluding quantitatively that this behavior was in fact due to a conserved sense of effort. Our findings, though inconclusive, suggest that the conventional form of cost function used is not sufficient for analyzing the optimality, or lack their of, of human motor behavior.

## Methods

### Experimental Protocol

The Institutional Review Board at the University of Colorado Boulder approved all experimental procedures. All participants were naïve to the goals of the experiment and were paid to participate. Eight young adults (20–26 years, 4 Males) participated in the experiment after providing written consent.

Subjects were instructed on a task designed to simulate walking along the edge of a cliff. By pushing/pulling against the handle of a force transducer, subjects’ accelerated/decelerated the vertical trajectory of a virtual mass as it moved steadily, horizontally toward a target on the far right end of the screen. In some trials, the middle of the screen represented the edge of a cliff, such that if the mass moved below it, it experienced a large downward acceleration, requiring subjects to produce large forces to recover the mass’ trajectory. In still other trials noise randomly perturbed the mass’ height, increasing the possibility it would slip over the edge of the cliff. Though the dynamics and uncertainty of the task conditions varied, the rewards for landing on target were always the same. Thus the task was designed to examine subject’s subjective tradeoff between the effort involved in guiding the mass and the potential rewards. Below we describe the experiment in more detail.

**Figure 1:**
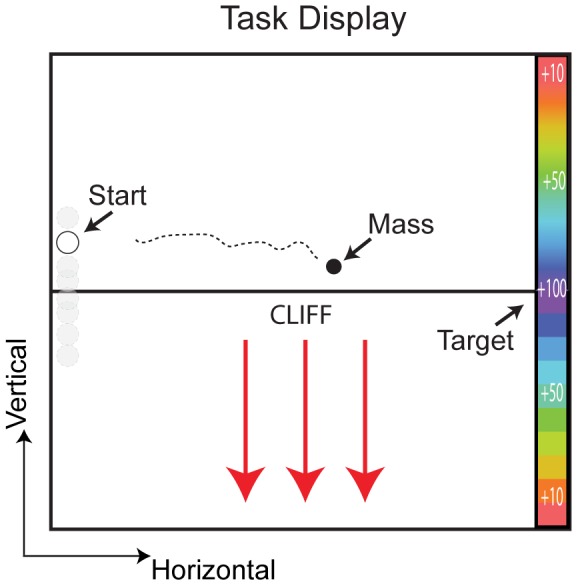
Experimental set up and display. Subjects were seated in front of a screen, holding a handle mounted to a force transducer. By pushing/pulling on the handle subjects accelerated/decelerated the vertical motion of a cursor that moved horizontally across the screen. On the right edge of the screen was a color-coded target that depicted rewards decreasing quadractically from the central target.

Subjects were seated in front of an LCD screen at approximate eye level (see Fig. 1) while grasping a handle rigidly mounted to a force transducer (JR3, 6-axis force-torque sensor). Each trial began with the cursor, representing a virtual mass, at the left edge of the screen. Vertical starting locations were drawn from a bimodal Gaussian distribution (modes at y = +/−2.5 cm, σ = 1 cm), such that trials could begin with the cursor either above or below the midpoint of the screen. The horizontal starting locations did not vary. After a brief pause, the cursor would then move horizontally across the screen with a constant velocity. Subjects controlled the cursor’s vertical movement by isometrically pushing or pulling on the handle. They were instructed to guide the cursor towards the vertical target at the center of the right edge of the screen (y = 0, depicted with a horizontal line across the screen (Fig. 1). The y-component of the force measured at the handle was used to accelerate the simulated cursor dynamics: a frictionless point mass (*m = *80 kg). Since the cursor’s horizontal velocity was constant, all trials had the same duration (approximately 1.7 seconds) and ended when the cursor reached the right edge of the screen. Points were awarded based on the cursor’s terminal position with respect to the target. A maximum of 100 points was awarded for hitting the target directly. Terminal positions off target were awarded points that decayed quadratically, such that the maximum target error (at the top or bottom of the screen) yielded 0 points. After each trial, subjects were informed of the points earned and a counter of their cumulative points was located in the upper right hand corner of the screen at all times. Force, and cursor position, velocity, and acceleration were recorded and updated at 200 Hz.

In some conditions (see below) the midpoint (y = 0) represented a virtual cliff; if the cursor (mass) moved below it, it experienced a virtual downwards force. To bring the cursor back towards the target once it had fallen below the cliff required relatively large forces on the part of the subject. Thus the virtual cliff induced an implicit penalty and nonlinearity in the cursor’s dynamics. In all settings the cursor’s vertical acceleration was simulated as:
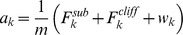
Where *a*, *F^sub^*, *F^cliff^*, *w* are the cursor acceleration, subject-applied force, nonlinear cliff force and additive noise, respectively. At each time step, *k*, the additive noise, *w_k_*, was drawn from a zero-mean Gaussian distribution with standard deviation σ.

Each subject visited the lab twice and took part in 6 different blocks: *NULL*, *CLIFF*, *NOISE-SMALL*, *NOISE-LARGE*, *CLIFF+NOISE-SMALL*, and *CLIFF+NOISE-LARGE*. In the linear *NULL* block, cliff dynamics and additive noise were not present (*F^cliff^ = *0, σ = 0). In the *NOISE* blocks, two different levels of noise were added to cursor acceleration in the vertical axis (σ_small_ = 75 m/s^2^, σ_large_ = 120 m/s^2^). These trials, though not analyzed here, familiarized the subjects with the noisy perturbations to the cursor. In the nonlinear *CLIFF* block, a nonlinear environment was simulated with a virtual cliff at the midline of the screen (*F^cliff^* = −65 N). If the cursor fell below the cliff, subjects had to exert significant effort to counter this additional force and return the cursor above the midline, where the cliff dynamics were not present. In the nonlinear stochastic *CLIFF+NOISE* blocks, both the noise (σ_small_ or σ_large_) and nonlinear cliff dynamics (*F^cliff^* = −65 N) were present.

On the first visit 5 subjects were tested in the *NULL*, *NOISE-SMALL*, *CLIFF*, and *CLIFF+NOISE-SMALL* blocks. On a second day of testing, they performed the remaining *NOISE-LARGE* and *CLIFF+NOISE-LARGE* blocks. For the remaining subjects the order in which they experienced the *NOISE* blocks was reversed. In each block subjects first performed 50 practice trials where points were not awarded. This was followed by a testing session, consisting of 300 trials in the same condition, for which points were tallied for their final score. During the practice trials, half started above the midline. In the testing sessions the order of trials starting above or below the midline/cliff was pseudo-randomized such that one in every 4 trials in the testing session started below the midline/cliff. Since our analysis focused on subjects’ behavior in trials starting above the cliff we wanted to maximize the number of these trials. However, trials that started below the cliff were still needed to ensure the subjects experienced the cliff dynamics. To maintain consistent initial conditions across trials, a trial only began when the forces exerted by the subject on the handle were negligible (|*F^sub^*|<0.25 N).

### Behavioral Analysis

Based on preliminary data we found that after approximately 100 trials, subjects converged to a steady behavior. Therefore only the last 200 successful trials in each block were examined. Successful trials were those where the cursor never moved off the screen (above or below the screen dimensions), and never fell below the cliff by more than 10 cm. By neglecting the 50 practice trials plus the additional first 100 test trials of each block we mitigate any learning effects that might skew our analysis. As we are interested in how subjects behave when there is the threat of encountering the cliff and the nonlinear change in dynamics, we limited our analysis of the cliff blocks to trials beginning above the cliff.

To compare the effects of the different conditions we used 4 outcome measures meant to characterize the influence of risk and uncertainty on subject behavior: the peak vertical cursor displacement from the cliff, the mean cursor position (above the cliff), and the peak positive and negative force values, during a trial. We performed one-way repeated-measures ANOVAs with block as a factor. Significance levels were set at α = 0.05.

### Model Analysis

To predict optimal behavior we first need to identify a set of variables and a dynamical model that describes how these variables evolve over time. Then, by inferring an approximate cost function in terms of these variables in the *NULL* block, we can predict his or her behavior in the subsequent settings. Below we explain this procedure in detail.

Each subject’s cost function approximated how he or she weighted the cursor’s motion, and a trial’s ultimate reward against the effort they employed. Effort has been quantified in many different ways including metabolic cost [20], [27], neural command [11], mechanical work [28], force generation [29], and rate of force generation [6], [10]. Based on practical considerations, and owing to the fact that subjects performed the task isometrically, we assumed effort could be quantified in terms of the handle force and its rate of change. Accordingly we chose to define a cost function in terms of the cursor’s position, velocity, and the subject’s handle force and its derivative, or symbolically, *y*, *v*, *F*, d*F*/d*t*.

Obtaining a dynamical model for these variables is straightforward. The cursor dynamics, simulated discretely during each trial, were known accurately. The relation between the handle force and its derivative is similarly straightforward. As such, the variables of concern could be defined through the state space dynamics,
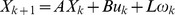
Where the state was defined as *X* = [*y*, *v*, *F*], and the command *u* as = d*F*/dt. The matrices were appropriately defined as *A* = [1, δ*t*, 0; 0, 1, δ*t*/*m*; 0, 0, 1], and *B* = [0; δ*t*; 0], where δt = 0.05 s. Allowing for the subject’s own variability, as well as the random perturbations during the noisy cliff conditions, noise terms were also modeled; *L* = [0; δ*t*; 0], and ω was assumed to be a Gaussian random variable, drawn from a zero-mean distribution with subject-specific standard deviation. The resulting discrete-time linear dynamical system could be used to quantify the optimal cursor and force trajectories for a given candidate cost function.

In general, a cost function for a motor control problem such as this would be expressed as the sum of two components. The first is an instantaneous cost that gets summed over all the time steps of the trial; for example, how hard the subject pushes against the handle at any moment. The second component is a terminal cost; for example, the mass’s final distance from the target. In the experiment, the task score was defined quadratically in terms of the cursor’s final distance from the target; subjects received the maximum score when they landed on target, *y* = 0, and a score/penalty that decreased/increased quadratically as the error increased (∝*y* ^2^). This was by design, since for reasons of mathematical convenience, and broad applicability, the two components of a cost function usually take the form of quadratic penalties,
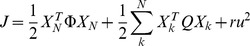
Where Φ is the penalty on the terminal state, *Q* is the instantaneous penalty for having non-zero position, velocity and force during the task, and *r* is the cost on the derivative of the force. While subjects only received points based on the final cursor position, this cost function allows for the possibility that subjects implicitly assign costs to other variables either during the trial or at its conclusion. For example, this form of cost function allows for terms such as, y*F*Q_1,3_. While subjects could be using a more complicated, higher-order cost function, this form can be viewed as a Taylor expansion of a higher-order cost function, expanded around the state *X* = 0. Similarly, subjects could weigh the effort in pushing or pulling against the handle differently. However, we assumed this was not the case, and that the effort of pushing with one unit of force was equivalent to the effort of pulling with one unit of force.

In general this cost function has 13 free parameters (the elements of the symmetric Φ, *Q,* and the scalar *r*) and the model dynamics have one free parameter (subject motor noise, ω). Since the problem is linear, and the cost quadratic, for any choice of these free parameters we could solve the associated value function and obtain the resulting optimal distribution of trajectories and their mean. To avoid over-fitting we only considered diagonal matrices for the cost function. Importantly, the terminal penalty for the cursor position, Φ_1,1_ was set to the actual value based on the point penalties explicitly imposed in the experiment. Since the penalty for the cursor’s position correctly corresponded with the rewarded points for the experiment, our approximate cost function can be viewed as a patient-specific sense of effort relative to the task’s point system. The remaining 7 free parameters were fit to each subject.

Minimizing the RMS error between subject data and the model, as well as a small regularizing term, these seven free parameters were fit to each subject. The RMS error term was computed in terms of mean cursor position, velocity, and force as well as their associated standard deviations in the *NULL* block. The use of a small regularizing term (here, 1E-7) is common practice in parameter estimation, penalizing non-zero parameter values and helping to establish a unique solution. Matlab’s constrained function minimizing routine, *fmincon*, was used to find these seven parameters. The resulting cost function was an approximate representation of how each subject weighed their effort relative to the true numeric score for the task. Importantly, this subject-specific fitting procedure was performed only once and only based on data in the *NULL* block, a linear, deterministic movement setting.

These subject specific cost functions were then used to predict optimal solutions in the subsequent settings. A discrete-time nonlinear stochastic optimal control algorithm [30] was used to approximate the subject-specific predicted optimal distribution of trajectories for each block. 5,000 simulated trials for each block were computed and used for quantitative comparisons with subject behavior. Using this approach we can compare not only the mean trajectories of the subject and model, but also trial-by-trial variability.

Just as with the subjects, for each model the four behavioral metrics were calculated. We determined the effect of movement setting on these metrics using a repeated measures ANOVA, similar to the analysis performed on the behavioral data. Additionally we performed three planned comparisons using paired t-tests on both the behavioral and model metrics to quantify differences between the following blocks: *CLIFF/NULL*; *CLIFF+NOISE-SMALL*/*CLIFF*; *CLIFF+NOISE-LARGE*/*CLIFF*. These tests assessed the predicted influence of movement setting on optimal behavior.

To specifically compare the subject and model results, a series of comparisons were made. For an overall quality of subject-specific fits, correlations (R) between subject and model were computed for each experimental block between the position profiles, as well as the combined correlation between the position, velocity, force, and their corresponding variability profiles. To compare behavioral metrics, we performed a linear regression between subject and model data; a slope of 1 would indicate a perfect correspondence. Finally, to determine whether individual model behavioral metrics matched (i.e. could not be distinguished) from the subject behavioral metrics, two sample t-tests were performed. The criteria for statistical significance was set at α = 0.05.

## Results

### Overview

Subjects (n = 8) guided a cursor towards a target in linear, nonlinear and nonlinear stochastic settings. Overall subjects had little difficulty with the task and were able to land the cursor close to the target on most of the trials. As the settings became increasingly difficult (linear, *NULL* to nonlinear, *CLIFF*, to nonlinear stochastic, *CLIFF+NOISE*), the number of successful trials decreased and subject strategies, evidenced by their movement and force trajectories, became distinct. The data from the linear setting was used to infer each subject’s cost function, quantifying the tradeoff between effort and reward. The cost functions were then used to predict each subject’s behavior in the subsequent settings, assuming they optimized that same subject-specific costs. The observed subject behaviors, quantified through several metrics, were compared with their model predictions to examine if they were consistent with the optimization of a single cost function.

### Linear Setting

**Figure 2:**
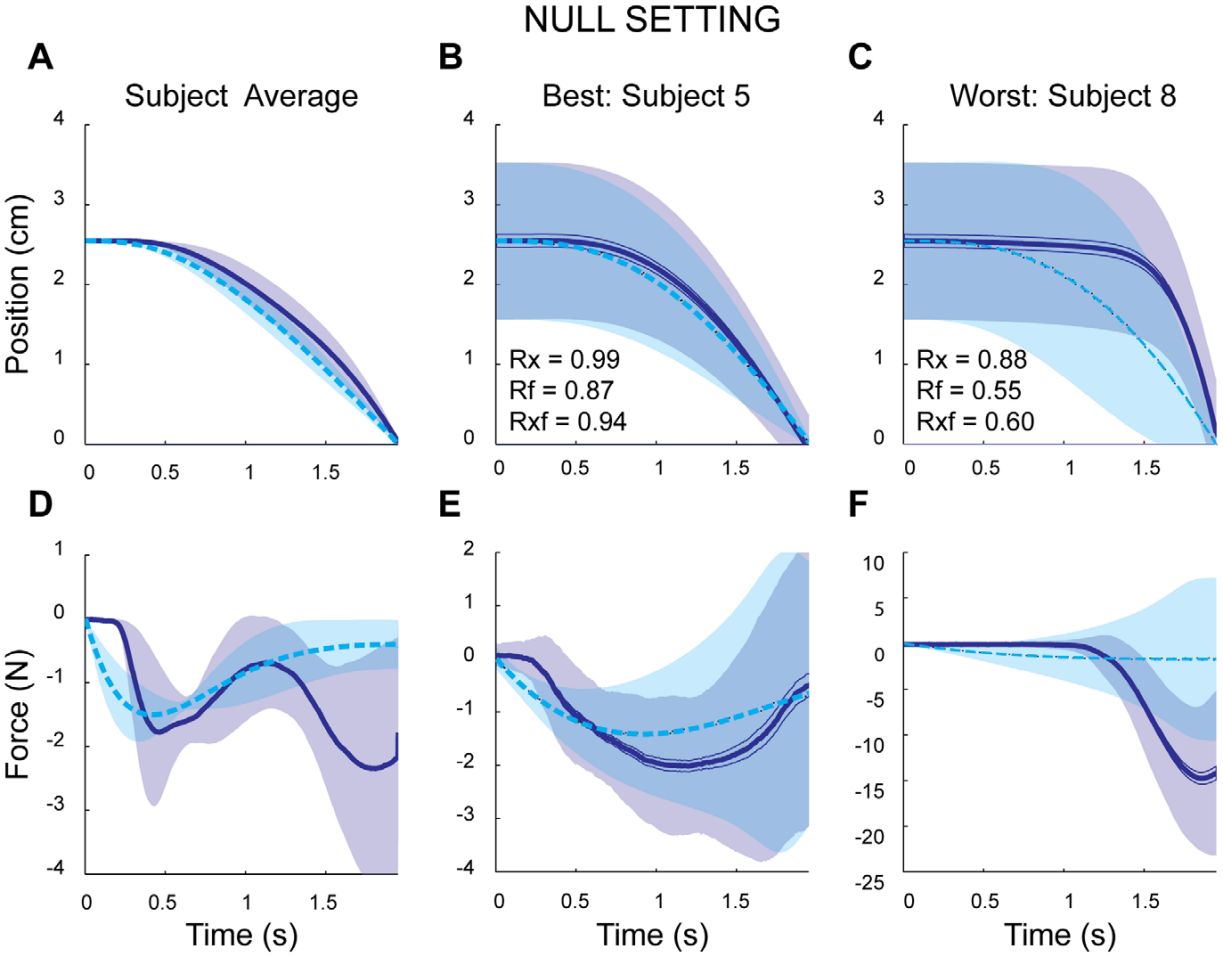
Example subjects and model data in the *NULL* block (velocity profiles not shown). A), D) Across-subject/model average trajectories for position and force, respectively. B), E) The subject and model with the best fit. C), F) The subject and model with the poorest fit. In all panels solid and dashed lines indicate subject and model mean trajectories, respectively. Shaded regions represent either +/− SEM (panels A and D) or +/− standard deviation across trajectories (remaining panels).

Subjects first completed the *NULL* block in the linear setting. This was done to familiarize them with the task, and to find a cost function that could account for their data. Subjects easily achieved the task in the linear setting, achieving an average score per trial of 99.95+/−0.06 (mean +/− standard deviation), indicative of approximately 1 *mm* of error. Subjects generally guided the cursor through a smooth path from its initial location towards the target. Trajectories starting above and below the mid point of the screen were qualitatively similar and symmetric on average (see Fig. 2 for examples); the peak forces (2.24 N+/−2.5 N, and −7.15 N+/−4.77 N) above and below the midline were also indistinguishable (paired t-test: p>0.5). This supported our assumption of symmetric costs for pushing and pulling in this context. Across subjects, peak distances above the midline were low (2.57 cm+/−0.02 cm), as was the mean distance (1.80 cm+/−0.38 cm), illustrating that subjects generally did not move further away from the midline than the original starting position.

The behavior during this setting was used to infer each subject’s cost function relative to the task’s scoring system. The model fits to this data were generally good, with small RMS errors and high correlations. The average correlation between subject and model cursor position was high (0.97+/−0.04). Similarly, the average correlation between subject and model when comparing the combined data of position and force profiles was high (0.82+/−0.09; Fig. 2). The model’s predicted behavioral measures were qualitatively similar to the subject metrics (peak distance: 2.63 cm+/−0.07 mean distance: 1.65 cm+/−0.28 cm; peak positive force: 2.46 N+/−1.97 N; peak negative force: −3.82 N+/−2.02 N). Overall, these results suggest our choice of variables and inferred weights produced a good approximation to individual subject motor costs.

### Nonlinear Setting

**Figure 3:**
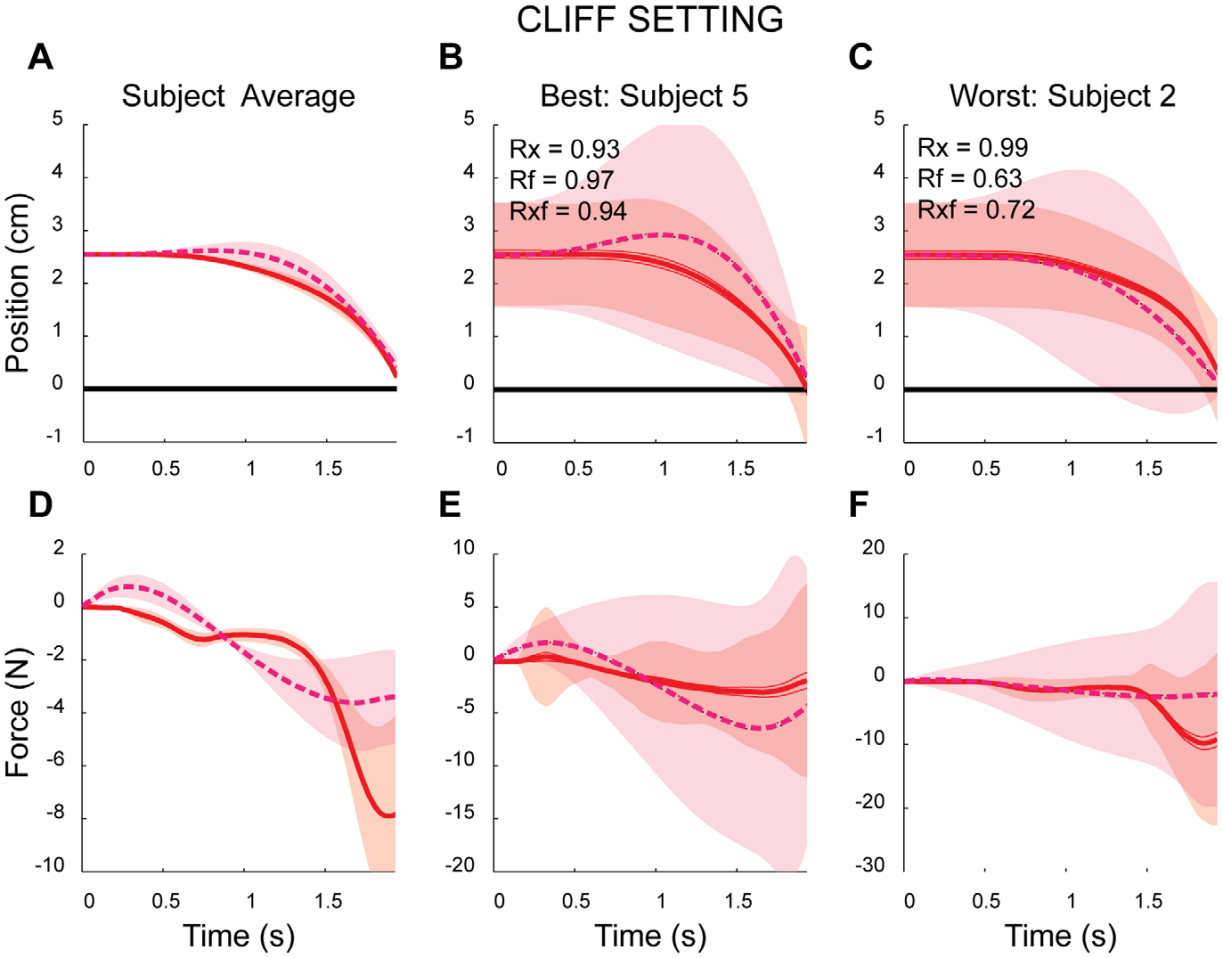
Example subjects and model data in the *CLIFF* block (velocity profiles not shown). A), D) Across-subject/model average trajectories for position and force, respectively. B), E) The subject and model with the best fit. C), F) The subject and model with the poorest fit. In all panels solid and dashed lines indicate subject and model mean trajectories, respectively. Shaded regions represent either +/− SEM (panels A and D) or +/− standard deviation across trajectories (remaining panels).

In the nonlinear CLIFF block, the task was no longer symmetric. In a deterministic sense, trajectories beginning above the cliff need not differ from the previous linear setting. However, owing to the subject’s inherent motor noise, we assumed their behavior would change. This would serve as our first test for the possibility that a single cost function generalized across different settings. Subjects had little difficulty with the task and were able to land the cursor close to the target on most of the trials. In trials where the cursor began above the cliff, subjects were either slow in forcing the cursor’s downward motion, or simply moved the cursor in the opposite direction, away from the cliff, initially. As a result the cursor remained relatively far from the cliff’s edge until the end of the trial, at which point the subjects forced it to quickly approach the target (see Fig. 3 for examples).

This change in the cursor’s trajectories was captured by the mean distance above the cliff in this setting (2.06 cm+/−0.19 cm) relative to the linear setting, (p = 0.02). The peak negative forces were also slightly greater in this setting (−12.10 N+/−9.38 N; p = 0.07). However, the peak distance above the cliff and peak positive force were similar between settings (2.60 cm+/−0.05 cm, 1.63 N+/−1.37 N; p = 0.19 and p = 0.42, respectively). These results indicate that on average subjects avoided the cliff for a longer time before forcing the cursor to the target.

To examine whether subjects appeared to be optimizing a conserved cost to produce these movements, we then used the subject-specific costs inferred from the linear setting to predict each subject’s behavior in this nonlinear setting. Subject behavior was qualitatively consistent with the optimal model predictions with high correlations for position profiles, as well as the combined correlation between position and force profiles (0.94+/−0.04, 0.77+/−0.12, respectively). Model behavioral metrics also exhibited similar trends to the subject data. The implicit risk of the cliff increased the peak distance from the cliff to 3.06 cm+/−0.42 cm, with a mean distance of 2.19 cm+/−0.39 cm. There were commensurate changes in the forces, with the peak maximum forces now 3.36 N+/−1.76 N, and the negative forces increased to −7.59+/−5.4 N. Model predictions exhibited small, yet significant differences between linear and nonlinear settings in peak distance, mean distance, and peak negative force (p = 0.014, 0.017 and 0.032, respectively). In accordance with subject behavior, peak positive force was similar between settings (p = 0.08).

### Nonlinear Stochastic Setting

Following the nonlinear setting, subjects performed the task in two versions of a nonlinear stochastic setting, the *CLIFF+NOISE-SMALL* block and the *CLIFF+NOISE-LARGE* block. The added stochasticity made avoiding the cliff more difficult and subjects would need to compensate for the block-specific noise properties if they were to avoid the cliff. These two blocks would serve as our second and third test for a conserved sense of effort.

### *CLIFF+NOISE-SMALL* Block

**Figure 4:**
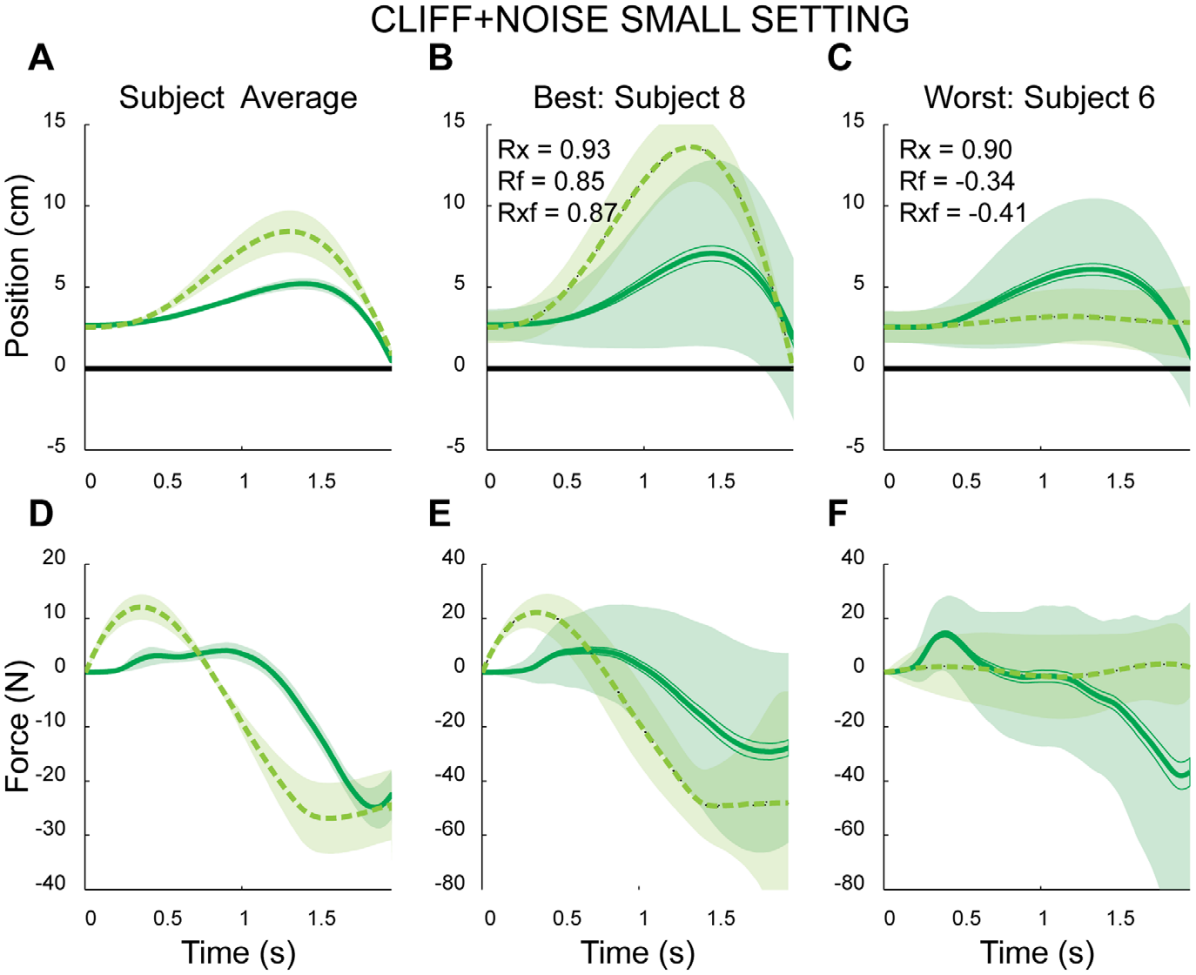
Example subjects and model data in the *CLIFF+NOISE SMALL* block (velocity profiles not shown). A), D) Across-subject/model average trajectories for position and force, respectively. B), E) The subject and model with the best fit. C), F) The subject and model with the poorest fit. In all panels solid and dashed lines indicate subject and model mean trajectories, respectively. Shaded regions represent either +/− SEM (panels A and D) or +/− standard deviation across trajectories (remaining panels).

Subjects altered their strategy relative to the previous settings by moving the cursor away from the cliff initially and only driving it towards the target during the final moments of each trial (Fig. 4). Endpoint error increased significantly to 5.5 mm +/−5.9 mm, compared to the *CLIFF* block (p = 0.04). In contrast to the transition from *NULL* to *CLIFF*, the transition from *CLIFF* to *CLIFF+NOISE-SMALL* led to differences in all four metrics (all p’s <0.001). The new peak displacements increased to 7.02 cm+/−0.81 cm with new mean distances of 3.73 cm+/−0.47 cm. The peak forces similarly increased/decreased to 26.19 N+/−8.73 N and −49.87 N+/−9.28 N. These differences made it clear that subjects’ behavior was influenced by the added noise, and in a manner consistent with a fear of the cliff.

Using the same cost functions inferred from the linear setting, the model predictions correlated well with subject behavior (0.88+/−0.13, 0.80+/−0.07). Model-predicted metrics exhibited similar trends as the subject data, with all four metrics increasing between *CLIFF* and *CLIFF+NOISE-SMALL* (all p’s <0.001). Here the across model average peak displacement increased to 8.94 cm+/−3.35 cm with a mean distance of 5.33 cm+/−1.73 cm. Average model peak forces were 16.99 N+/−4.85 N and −39.12 N+/−21.68 N.

### *CLIFF+NOISE-LARGE* Block

**Figure 5:**
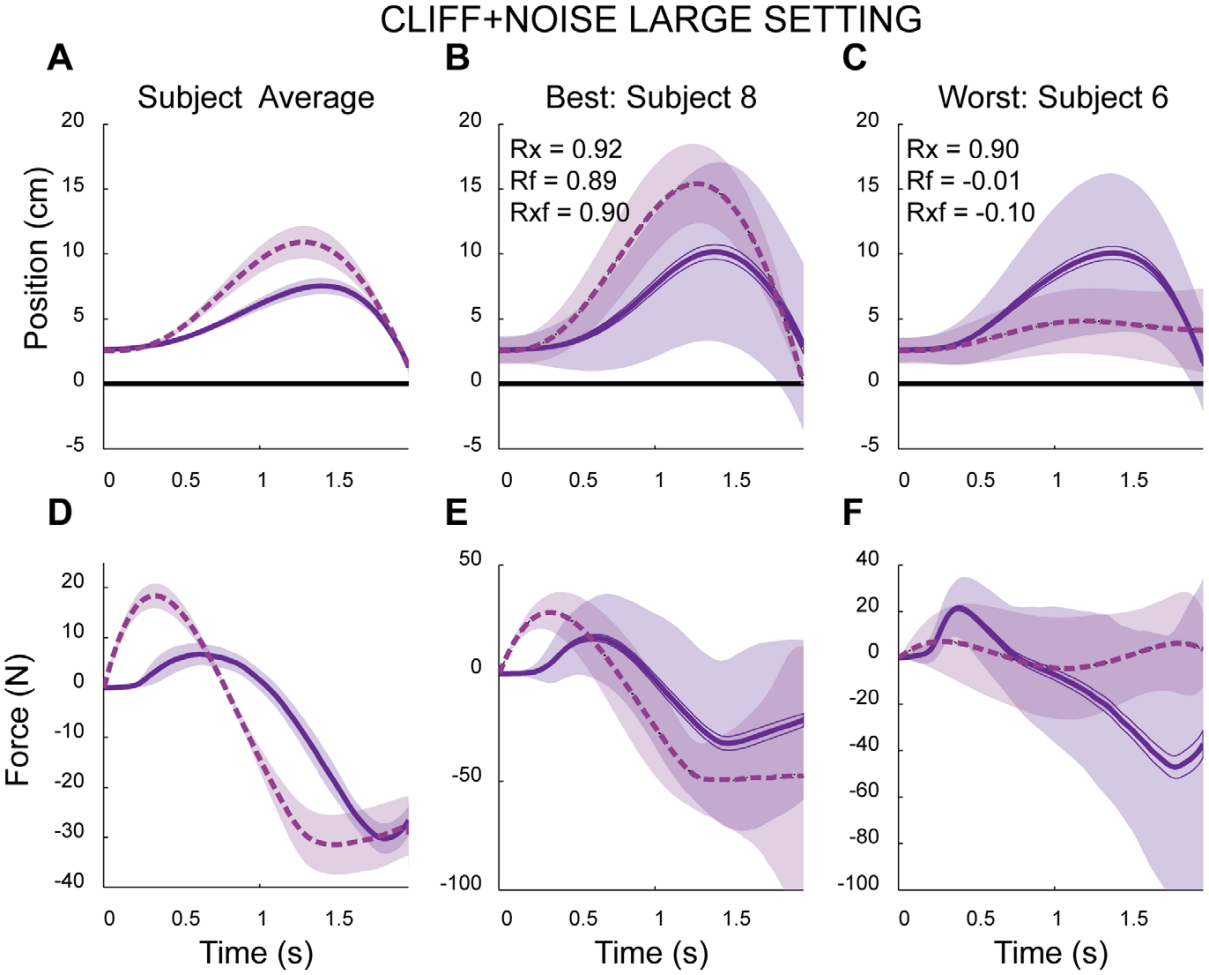
Example subjects and model data in the *CLIFF+NOISE LARGE* block. A), D) Across-subject/model average trajectories for position and force, respectively. B), E) The subject and model with the best fit. C), F) The subject and model with the poorest fit. In all panels solid and dashed lines indicate subject and model mean trajectories, respectively. Shaded regions represent either +/− SEM (panels A and D) or +/− standard deviation across trajectories (remaining panels).

If subjects were optimizing a cost sensitive to the risks of noise, then increasing the noise would lead to changes in behavior. In this block of trials, the experimentally introduced noise was nearly doubled. This increased the difficulty of the task and average endpoint error increased to 13.5 mm+/−8.4 mm, significantly greater than the error observed in *CLIFF+NOISE-SMALL* setting (p = 0.012). There were changes in the behavioral metrics as well. The across subject peak displacement was 10.0 cm+/−1.25 cm with a mean distance of 4.99 cm+/−0.87 cm. The across subject average peak forces were 31.87 N+/−11.96 N and −59.45 N+/−11.25 N. Compared to the *CLIFF* block (deterministic), subjects in *CLIFF+NOISE-LARGE* block avoided the cliff to a greater degree and used greater forces (see Fig. 5, all p’s <0.001). These differences were also evident, albeit to a lesser degree, when behavior was compared to the *CLIFF+NOISE-SMALL* block (p = 0.0001, 0.0007, 0.158, 0.0069, respectively). Thus, the observed behavior was clearly influenced by the level of noise.

Finally, subject cost functions were used to compute model predictions. These predictions resulted in high correlations with subject behavior when compared against the position profiles, and the combined position and force profiles (0.95+/−0.02, 0.64+/−0.32, respectively). Just as with the subjects, the optimal models demonstrated changes in behavior and moved away from the cliff. The new across model peak displacement was 11.68 cm+/−3.11 cm with a mean distance of 6.69 cm+/−1.63 cm. The average model peak forces were 26.08 N+/−5.29 N and −51.75 N+/−22.29 N. Model-predicted metrics also exhibited significant increases in distance from the cliff and force compared to model-predicted metrics in the *CLIFF+NOISE-SMALL* block (all p’s <0.001 for peak distance, mean distance and peak forces). Once again, the high correlations and similar trends between subjects and their models were at odds with the clear differences between their trajectories (Fig. 5).

### Comparison of Subject Behavior and Model Predictions

The results thus far have demonstrated that subject behavior and subject-specific model predictions follow the same trends. Specifically, as the movement settings changed from linear, to nonlinear, to nonlinear stochastic, the risk of the virtual cliff elicited increases in peak and mean distance from the cliff, as well as peak maximum and minimum forces. These changes were statistically significant for both subjects (rmANOVA, all p’s <0.0001) as well as models (rmANOVA, all p’s <0.0001). Additionally, across all movement conditions, there were high average correlations between subjects’ trajectories and their model predictions. Thus the experimental conditions evoked behavior from the subjects and their models that was distinct and well correlated across settings.

Despite the consistent trends, there were often clear and qualitative departures between subject data and model predictions (see Fig.’s 3, 4, 5). Note that while the subject and model often had similar cursor trajectories, the velocity (not shown) and force profiles often differed considerably. For instance, though the correlations between subject and model trajectories were relatively large, they consistently (though not significantly) decreased as the movement settings became increasingly challenging. Similarly, the RMS error between subject and model trajectories significantly increased (p<0.001) as the movement settings became more challenging. These findings called into question our ability to accurately assess subject specific value functions and optimal behavior.

To further examine whether subject behavior was consistent with minimizing a conserved sense of effort, we directly compared model and subject behavioral metrics in each block using two-sample t-tests. Despite the often-high correlations, every subject was significantly distinct from their model in at least one behavioral metric, in each movement setting. With only one exception, each subject’s metrics became increasingly distinct from their model as the settings became more challenging. To illustrate, across the 4 tests for the 8 subjects there were 19, 25, 29 and 27 failed tests (out of 32) in the *NULL*, *CLIFF*, *CLIFF*+*NOISE-SMALL* and *CLIFF*+*NOISE-LARGE* blocks, respectively. Thus, on the whole, there wasn’t a single subject that was consistently indistinguishable from their model predictions across all settings.

**Figure 6:**
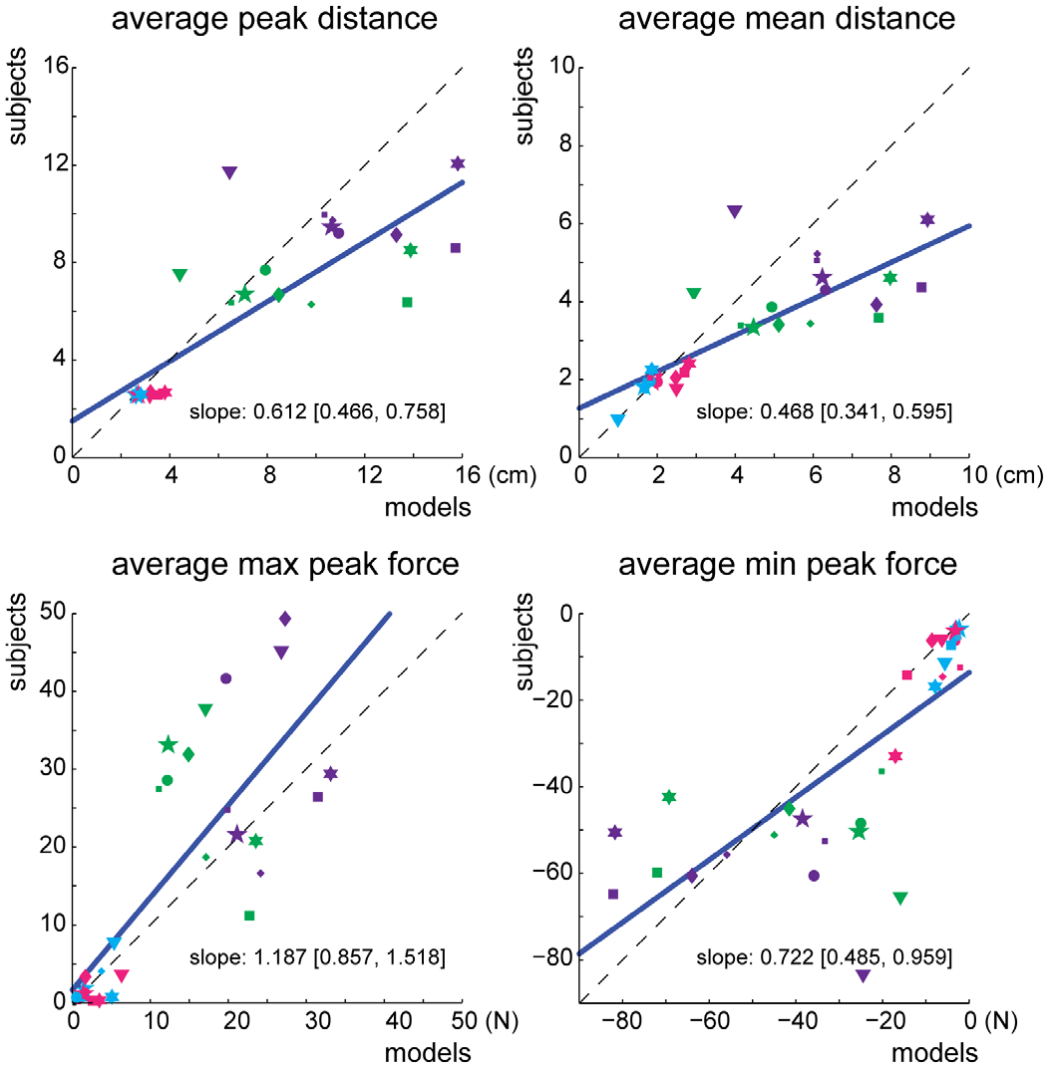
The four averaged behavioral metrics (peak vertical displacement, mean cursor position, peak positive and negative forces) of individual subjects plotted against their model predictions. The dashed line represents a one-to-one relationship between subject behavior and model predictions while the blue line is the least squares regression fit to the data. The slope and 95% confidence interval for each fit are displayed in the plots. Individual subjects are depicted with various shapes and the four blocks are color-coded (*NULL*: light blue, *CLIFF*: pink, *CLIFF*+*NOISE-SMALL*: green, and *CLIFF*+*NOISE-LARGE*: purple).

As a further examination, each subject’s average behavioral metrics were regressed against their model predictions to examine the trend across settings; a slope of unity would denote a perfect correspondence (Fig. 6). With the exception of peak positive force, a slope of unity fell outside the 95% confidence interval of all regressions. Taken together, our results suggest that while subjects behavior shared many qualitative features with what is optimal given our inferred subject-specific costs, we could not conclude that they were in fact optimal according to a conserved cost function.

## Discussion

Here we examined whether or not motor behaviors under different circumstances could be explained as the result of optimizing a single cost. Subject-specific costs were fit to a linear motor task, allowing us to interpret the behavior in terms of an optimized criterion. To validate these cost functions, we then had subjects perform the same task in nonlinear and nonlinear stochastic settings. In these settings subject behavior changed in small but distinct manners consistent with the added risk of the cliff and noise. Though these changes followed similar trends as their optimal predictions, they were distinct in many regards. As such we could neither conclude subjects used a conserved sense of effort, nor rule out this possibility. What we could conclude is that the conventional, and often used, quadratic penalty was inadequate to critically evaluate optimality. Ultimately our results indicate that a critical evaluation of the optimal motor control paradigm may require mathematical and experimental techniques more sophisticated than those commonly employed in the motor control field.

Given that the model predictions and subject behavior were often statistically distinct, we could not rule out the possibility that motor behaviors are optimizing a single cost function. Assuming that subjects actually did use a conserved sense of effort to generate behaviors then, broadly speaking, there are two possible reasons for this discrepancy: either the subjects are “wrong,” or the model is.

On the one hand, the subjects might have been performing sub-optimally. For example, it could be that subjects were not practiced enough at the task to reflect their optimal policies. The addition of nonlinearities and increased variability may also necessitate further practice before subjects could correctly arrive at their final policy. Additionally, we note that relative to many motor control studies, subjects in our task were producing large forces (regularly in excess of 60 N). Eliciting a broad range of varying responses from subjects was a benefit for the experimental paradigm, though it may have increased the difficulty of the task.

Given these considerations it is conceivable that significant amounts of experience with a motor task are necessary before subjects arrive at a behavior that correctly satisfies their inherent costs. Indeed, this may be a reason for the difference between novices and experts in other demanding motor tasks such as those used in team and competitive sports [31]. However, we provided each subject with many practice trials (150), and multiple days on the task. Moreover, a preliminary study on the same task found that subjects’ trajectories converged to stereotyped patterns after approximately 100 trials. This suggests that subjects were adequately practiced on the task. However, recent evidence has found that motor learning may persist even while movements remain invariant [27].

On the other hand, it could be that subjects were in fact behaving optimally and the model is wrong. This would imply that the fit cost functions are poor approximations to the subject’s true motor costs. This would not be surprising given that many other cost functions, not obviously consistent with the ones used here, have been argued to be the causes of motor behaviors. For instance, the subject’s true cost functions might include higher-order terms and variables not modeled (e.g. better indicators of effort and metabolic cost [20], [27]). Even in the *NULL* condition, where the cost functions were fit to the data, there were many significant differences between the subjects and their models. This implies that a quadratic cost function is not always adequate to capture subject behavior even in a linear, noise-free setting.

This conclusion has important implications for other studies that examine motor behavior in the optimal control framework, since the cost function we’ve used here, despite accurately modeling the true costs of the task, is the eminent form. Indeed, the form used here can recover both minimum jerk, torque rate and conventional motor command costs [5], [6], [10], [11]. Importantly, the typical quadratic cost assumes penalties on commands and states are additive. Future cost functions may investigate not only higher-order terms, but cross-terms as well. This is work we are currently pursuing.

The model could also be wrong in the assumption of a single cost function. The experiment was designed to control for many external factors such that a single cost function should generalize to the different settings. Based on previous studies and conceptualizations of motor control, there is no obvious reason why the cost function in the linear setting would not generalize to the other settings if the subject’s sense of effort didn’t generalize. However, it could be the case that subjects simply do not use a single cost function. Instead, different behaviors could use different cost functions, or similarly, cost functions might evolve as subjects become more familiar with a task.

This possibility too has important implications for motor control. If people choose their movements based on a cost function, but this cost function changes over time, and with behaviors, then future studies will require new innovative techniques to probe motor behavior and examine in which way it is optimal. Indeed, this may render an already difficult mathematical treatment of motor behavior intractable. Finally, perhaps most nihilistically, it could be the case that there is no cost function involved in the choice of motor behaviors [32]–[34]. Here again, researchers would have to consider new approaches to examining motor control.

It is worth noting that, theoretically, all behaviors are potentially optimal. That is, for any single motor behavior, there exists at least one criterion for which it is an optimal solution: the degenerate cost function, minimize deviations from the observed behavior. The existence of multiple optimal criteria is a potential hazard for all endeavors that attempt to examine the optimality of motor control. With this in mind, questioning the optimality of motor behaviors is less meaningful than asking what the motor costs are. While the quadratic cost function we have analyzed here was inadequate for critically evaluating optimality, it can be used for the more pragmatic undertaking of model comparison. For example, candidate cost functions that only penalize terminal errors, or only motor effort, can be used to generate optimal predictions. Statistical tests such as BIC, AIC and Bayes’ factor can then be used to compare the relative evidence of these different costs to explain the data. Successive model comparisons could then be used to refine what are more likely (and unlikely) costs. This approach would trade the search for the true cost function for an arbitrarily precise description.

An important contribution of this work is how we address the generalizability of motor costs across settings of varying dynamics. In examining whether or not motor costs generalize, we are questioning the validity of an approach used in many optimal studies of motor behavior. In these studies, a cost function that fits the data overall is argued to be the underlying cause of a motor behavior. Implicit in this proposition is the assumption that this cost function can predict motor behaviors in other settings as well. If not, the cost function and optimal predictions amount to little more than a sophisticated exercise in curve fitting. Here we have attempted to rigorously test the assumed conserved cost functions on a subject-specific basis. By fitting the optimal models with training data, and then validating these models with distinct data sets, we have avoided the problems of over-fitting, and more importantly, used a stringent standard for claims of optimality. Future studies investigating the existence of optimality strategies in motor control might consider similar requirements to move from the assumption of optimality to a rigorous examination.
